# Supplementary material for: BRCA1 and BRCA2 tumor suppressors protect against endogenous acetaldehyde toxicity
Source: EMBO Mol Med. 2017 Jul 20;9(10):1398–414. doi: 10.15252/emmm.201607446 (PMC5623864; doi:10.15252/emmm.201607446)
Supplement: Supplementary file 1 — Appendix [file EMMM-9-1398-s001.pdf]

BRCA1 and BRCA2 tumor suppressors protect against endogenous acetaldehyde toxicity

Eliana M. C. Tacconi, Xianning Lai, Cecilia Folio, Manuela Porru, Gijs Zonderland, Sophie Badie, Johanna Michl, Irene Sechi, Mélanie Rogier, Verónica Matía García, Ankita Sati Batra, Oscar M. Rueda, Peter Bouwman, Jos Jonkers, Anderson Ryan, Bernardo Reina-San-Martin, Joannie Hui, Nelson Tang, Alejandra Bruna, Annamaria Biroccio and Madalena Tarsounas

Table of contents

Appendix Figure S1.	Effect of olaparib, cisplatin, acetaldehyde and disulfiram on the survival of BRCA2-proficient and –deficient human DLD1 cells.	Page 1
Appendix Figure S2.	Acetaldehyde treatment reduces viability of BRCA1-deficient, but not 53BP1-deficient mouse cells.	Page 2
Appendix Figure S3.	Effect of olaparib, cisplatin and acetaldehyde on the survival of RAD51-proficient and –deficient human DLD1 and H1299 cells.	Page 3
Appendix Figure S4.	ALDEFLUOR™ assay in *Aldh2*$^{+/+}$ and *Aldh2*$^{-/-}$ MEFs.	Page 4
Appendix Figure S5.	Effect of acetaldehyde on the survival of mouse tumor-derived cell lines.	Page 5
Appendix Table S1 & S2.	Number of fiber tracks quantified in Fig 3.	Page 6

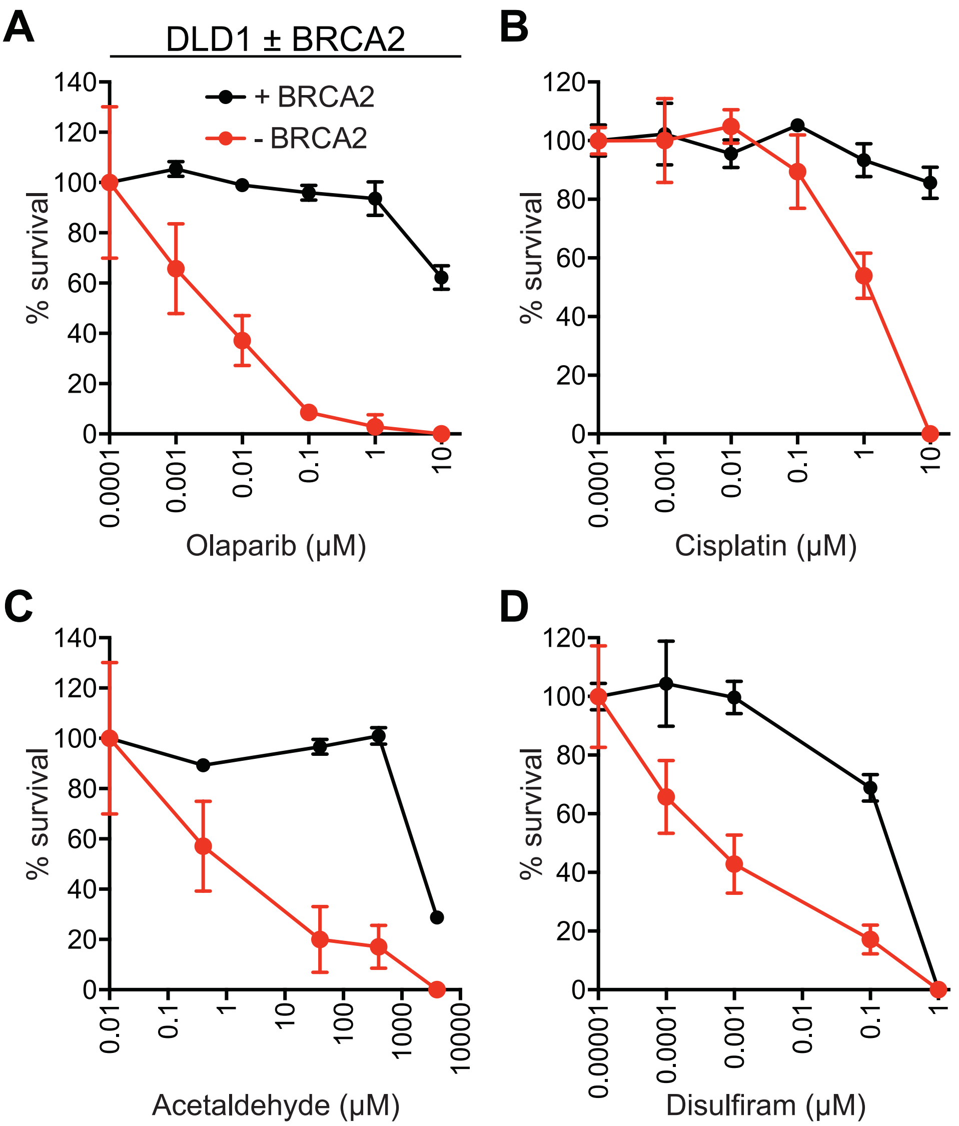

Appendix Figure S1. Effect of olaparib, cisplatin, acetaldehyde and disulfiram on the survival of BRCA2-proficient and -deficient human DLD1 cells.
Clonogenic survival assays of BRCA2-proficient (+BRCA2) or -deficient (-BRCA2) human DLD1 cells treated with the indicated concentrations of olaparib (**A**), cisplatin (**B**), acetaldehyde (**C**) or disulfiram (**D**) for 24 hours. Following removal of the drugs, cells were incubated in fresh media for 10-14 days before colony staining. Graphs are representative of two independent experiments, each performed in triplicate. Error bars represent SD of triplicate values obtained from a single experiment.

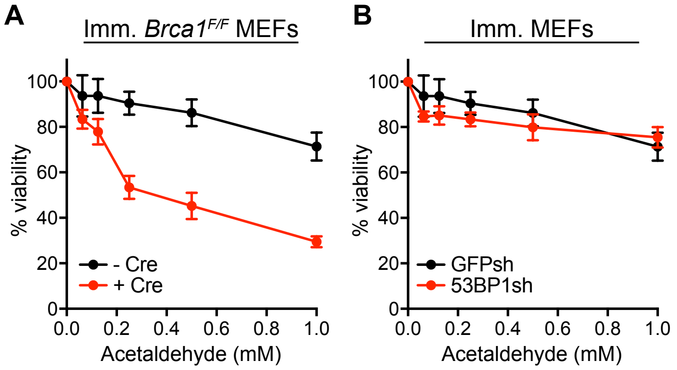

Appendix Figure S2. Acetaldehyde treatment reduces viability of BRCA1-deficient, but not 53BP1-deficient mouse cells.
A. Dose-dependent viability assays of *Brca1*$^{F/-}$ MEFs treated with Cre (+Cre) and control (-Cre) retroviruses. Four days post-selection, acetaldehyde was added to the media at the indicated concentrations and treatment was continued for six days. Graphs are representative of two independent experiments, each performed in triplicate. Error bars represent SD of triplicate values obtained from a single experiment.
B. Dose-dependent viability assays of immortalized MEFs infected with retroviruses encoding GFP control or 53BP1 shRNAs. Acetaldehyde treatment was performed as in (**A**). Imm, immortalized.

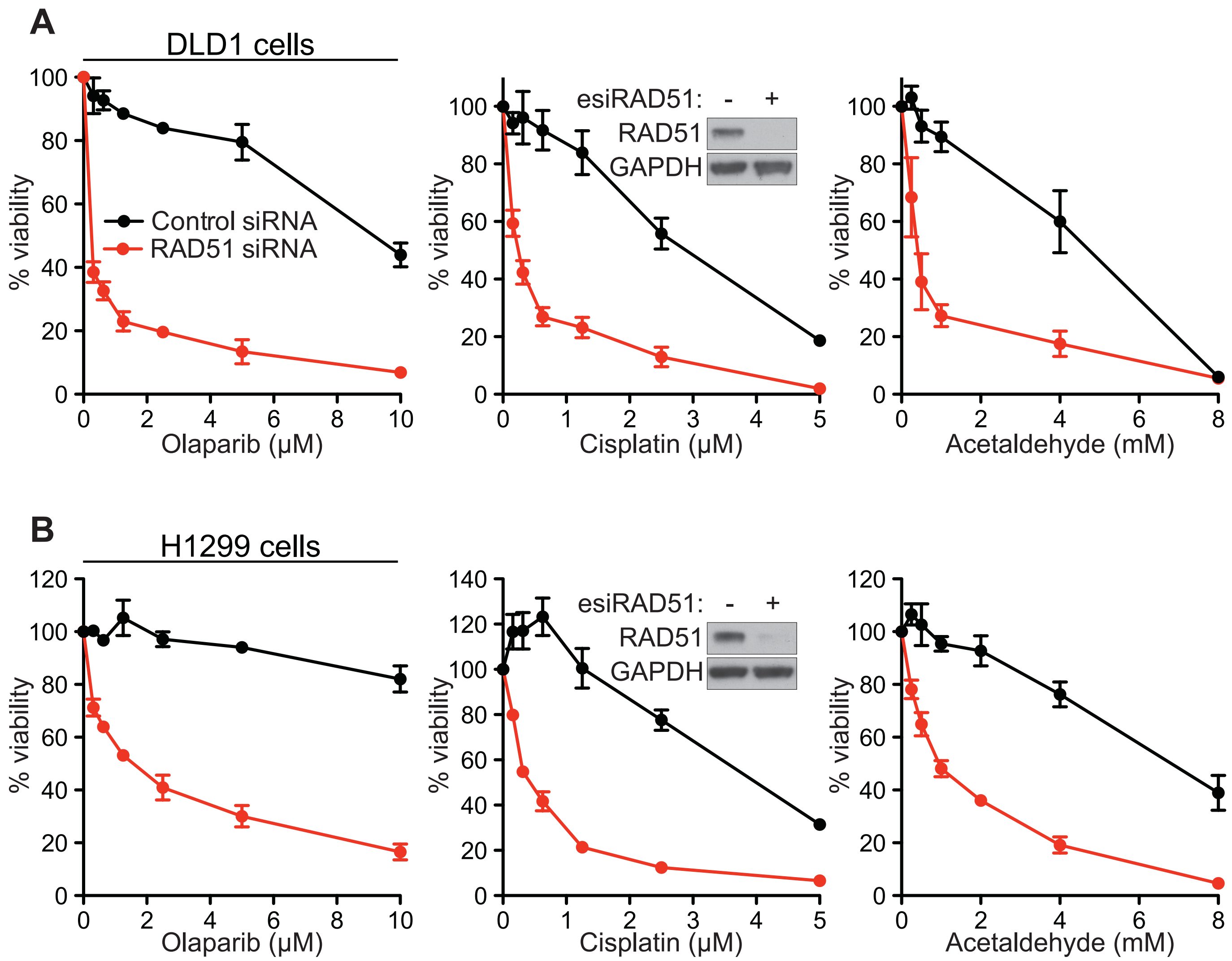

Appendix Figure S3. Effect of olaparib, cisplatin and acetaldehyde on the survival of RAD51-proficient and -deficient human DLD1 and H1299 cells.
Human DLD1 (**A**) and H1299 (**B**) cells were transfected with control or RAD51 esiRNA. Two days after transfection, olaparib, cisplatin or acetaldehyde was added to the media at the indicated concentrations and cells were incubated for six days before processing for dose-dependent viability assays. Graphs are representative of at least two independent experiments, each performed in triplicate. Error bars represent SD of triplicate values obtained from a single experiment.

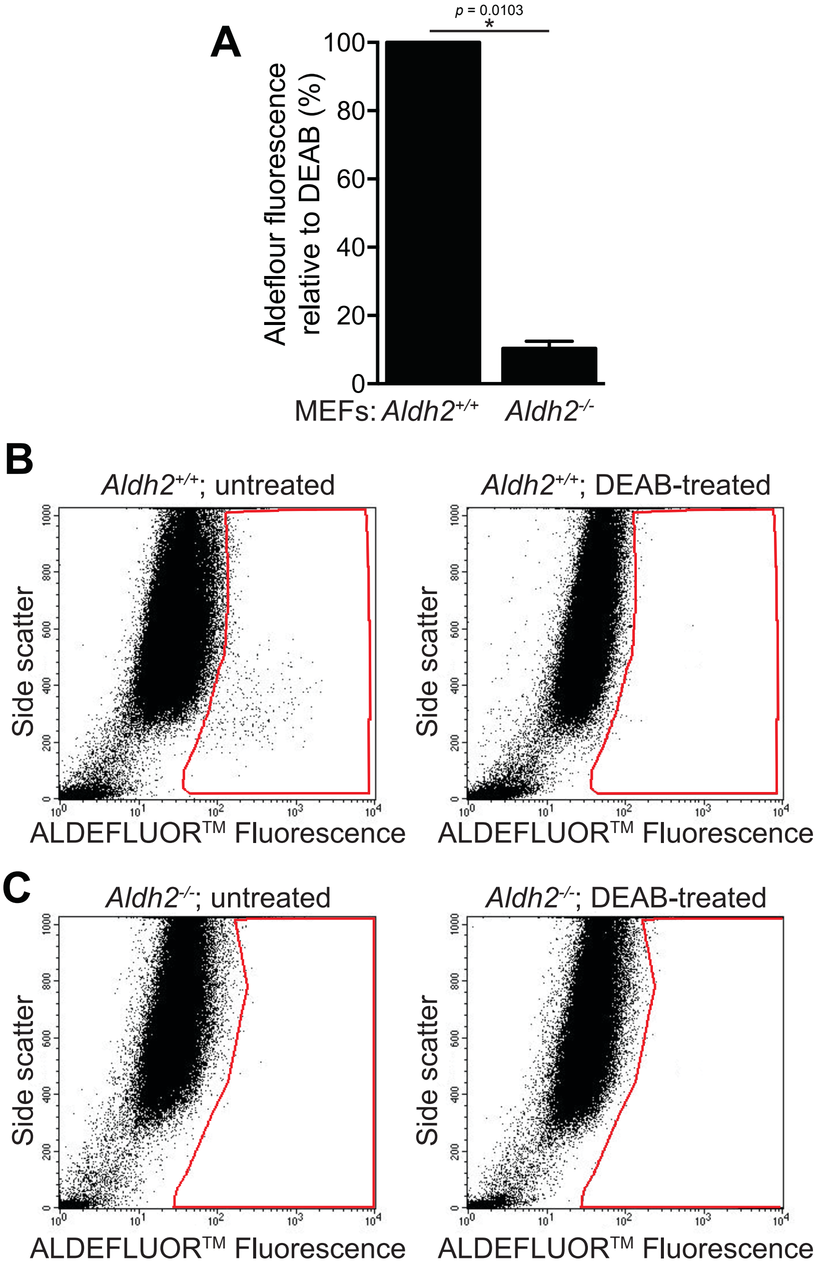

Appendix Figure S4. ALDEFLUOR™ assay in *Aldh2*$^{+/+}$ and *Aldh2*$^{-/-}$ MEFs.

A. Quantification of ALDH activity relative to internal DEAB control in *Aldh2*$^{+/+}$ and *Aldh2*$^{-/-}$ MEFs. Error bars represent SD of two independent experiments. *P* values were calculated using a one-sample *t*-test. *, $P \leq 0.05$.

B,**C.** Representative flow cytometry profiles in *Aldh2*$^{+/+}$ and *Aldh2*$^{-/-}$ MEFs. The general ALDH inhibitor DEAB is used as an internal control for background fluorescence. Red boundaries indicate cells with high ALDH levels.

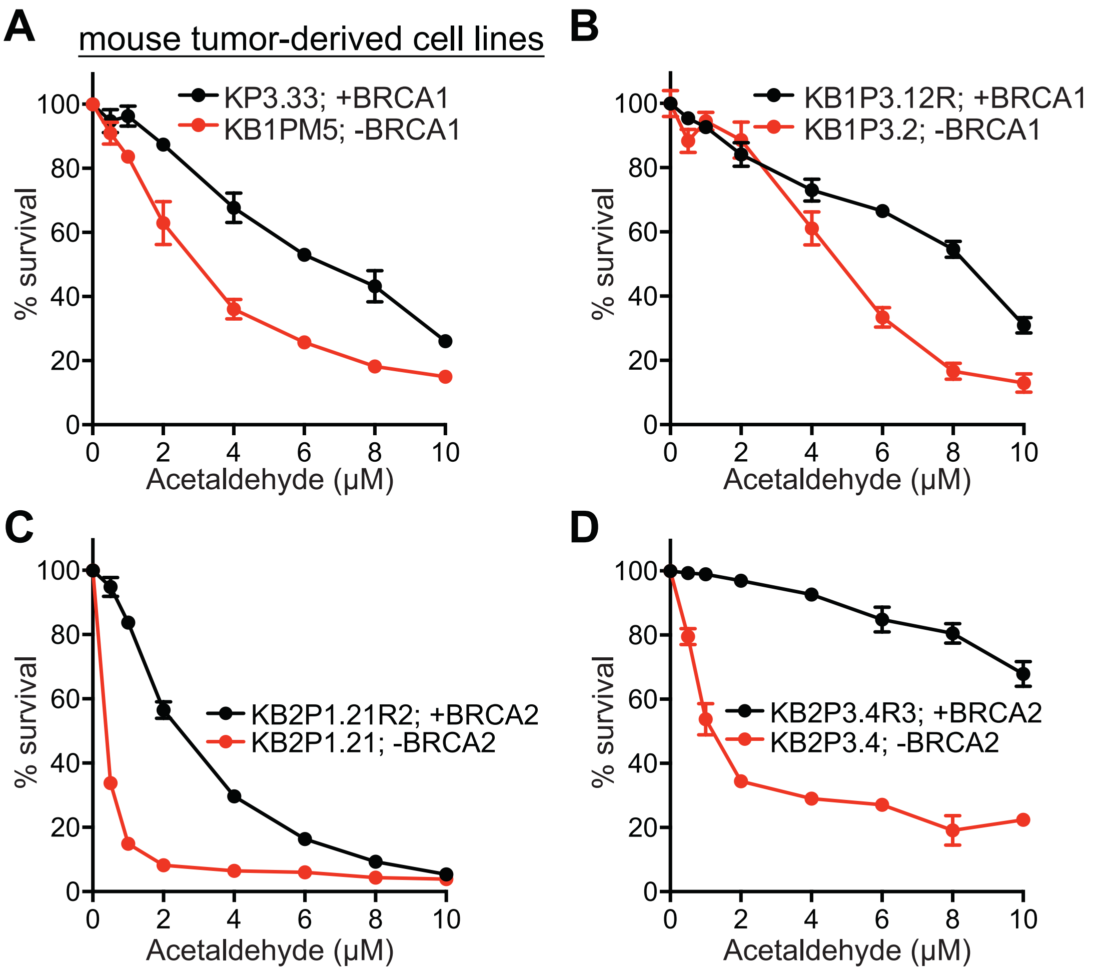

Appendix Figure S5. Effect of acetaldehyde on the survival of mouse tumor-derived cell lines.

Clonogenic survival assays of BRCA1-deficient (**A**,**B**) and BRCA2-deficient (**C**,**D**) mouse mammary tumor-derived cell lines treated with the indicated concentrations of acetaldehyde for 24 hours. Following removal of the drugs, cells were incubated in fresh media for 10-14 days before colony staining. Graphs are representative of three independent experiments, each performed with six technical replicas. Error bars represent SD of six technical replica values obtained from a single experiment.

Appendix Table S1. Number of fiber tracks quantified in Fig 3C.

	Treatment	Total number of fibers quantified
+BRCA2	Untreated	407
	Disulfiram	407
	Acetaldehyde	407
-BRCA2	Untreated	407
	Disulfiram	407
	Acetaldehyde	407

Appendix Table S2. Number of fiber tracks quantified in Fig 3D.

			Total number of fibers quantified
-DOX	-siFANCD2	-mirin	540
		+mirin	434
	+siFANCD2	-mirin	418
		+mirin	429
+DOX	-siFANCD2	-mirin	478
		+mirin	457
	+siFANCD2	-mirin	535
		+mirin	516
