# Supplementary material for: BRCA1 and BRCA2 tumor suppressors protect against endogenous acetaldehyde toxicity
Source: EMBO Mol Med. 2017 Jul 20;9(10):1398–414. doi: 10.15252/emmm.201607446 (PMC5623864; doi:10.15252/emmm.201607446)
Supplement: Supplementary file 2 — Expanded View Figures PDF [file EMMM-9-1398-s002.pdf]

Expanded View Figures

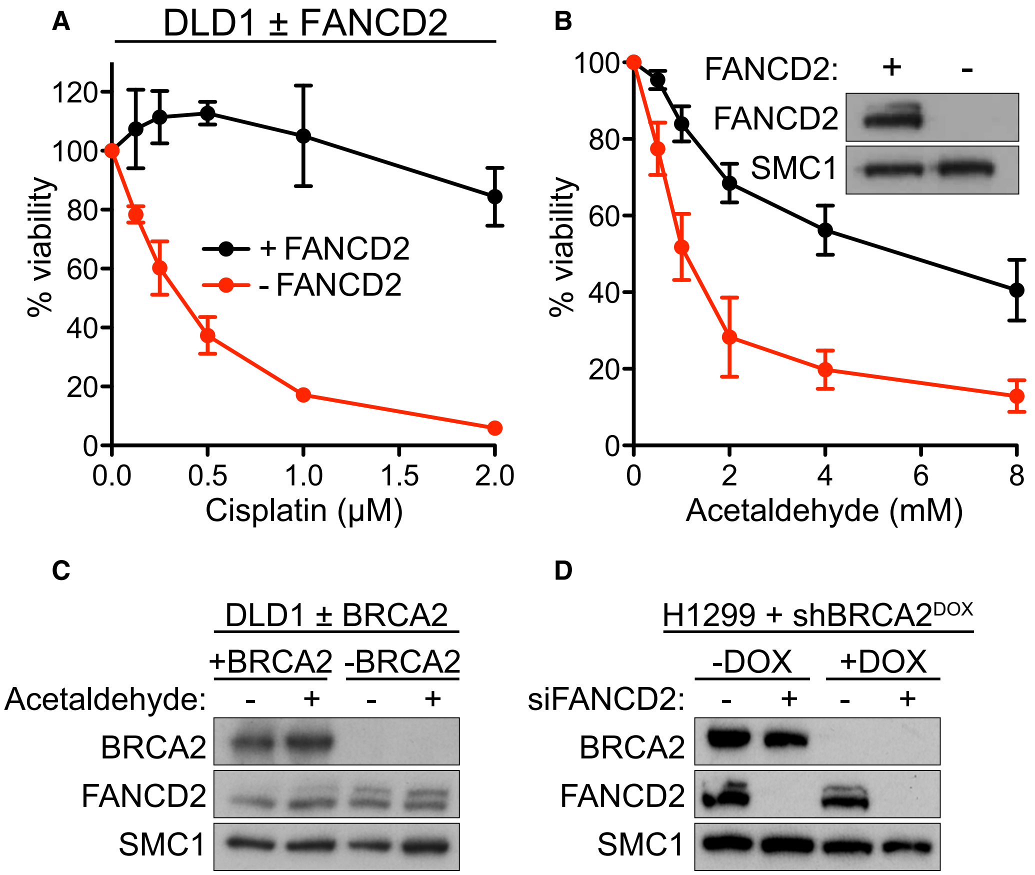

Figure EV1. Acetaldehyde toxicity to human FANCD2-deleted human cells and FANCD2 ubiquitylation in BRCA2-deleted cells.

A, B Human DLD1 cells in which FANCD2 was deleted with CRISPR/Cas9 and control cells were incubated with the indicated concentrations of cisplatin (A) or acetaldehyde (B) for 6 days before processing for dose-dependent viability assays. Graphs are representative of two independent experiments, each performed in triplicate. Error bars represent SD of triplicate values obtained from a single experiment. Inset, Western blot detection of FANCD2 expression. SMC1 was used as a loading control.

C BRCA2-proficient (+BRCA2) or BRCA2-deficient (−BRCA2) DLD1 cells were incubated with 4 mM acetaldehyde for 48 h before being processed for immunoblotting as indicated.

D H1299 cells expressing a DOX-inducible BRCA2 shRNA were grown in the presence or absence of DOX and transfected with control or FANCD2 siRNA before being processed for immunoblotting as indicated. DOX, doxycycline.

 © 2017 The Authors

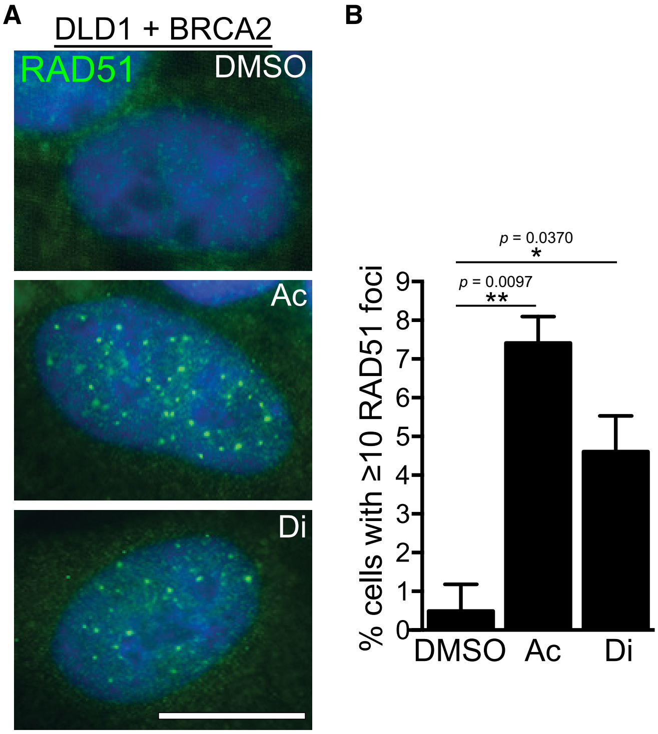

Figure EV2. Acetaldehyde and disulfiram induce RAD51 foci in BRCA2-proficient DLD1 cells.

A Human DLD1 cells, BRCA2-proficient (+BRCA2) were incubated with acetaldehyde (4 mM) or disulfiram (10 μM) for 96 h prior to processing for immunofluorescence staining with anti-RAD51 antibody (green). DNA was counter-stained with DAPI (blue). Scale bar, 10 μm.

B Quantification of percentage of cells with 10 or more RAD51 foci in cells treated as in (A). At least 100 nuclei were quantified for each treatment. Ac, acetaldehyde; Di, disulfiram. Error bars represent SD of two independent experiments. *P*-values were calculated using an unpaired two-tailed *t*-test.

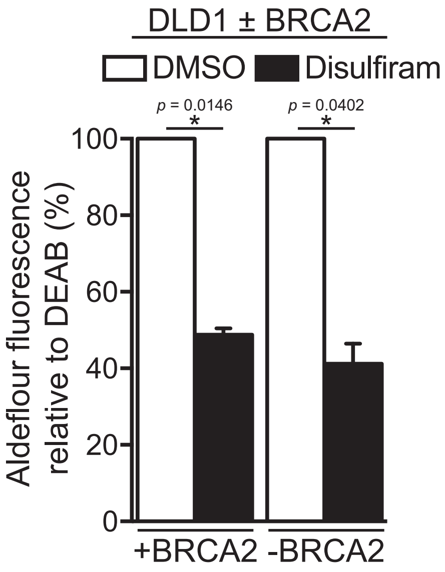

Figure EV3. ALDEFLUOR™ assay in human DLD1 cells treated with disulfiram.

Quantification of ALDH activity relative to internal DEAB control in BRCA2-proficient (+BRCA2) and BRCA2-deficient (−BRCA2) human DLD1 cells treated with DMSO or disulfiram (10 μM) for 4 days. Error bars represent SD of two independent experiments. *P*-values were calculated using a one-sample *t*-test.

© 2017 The Authors

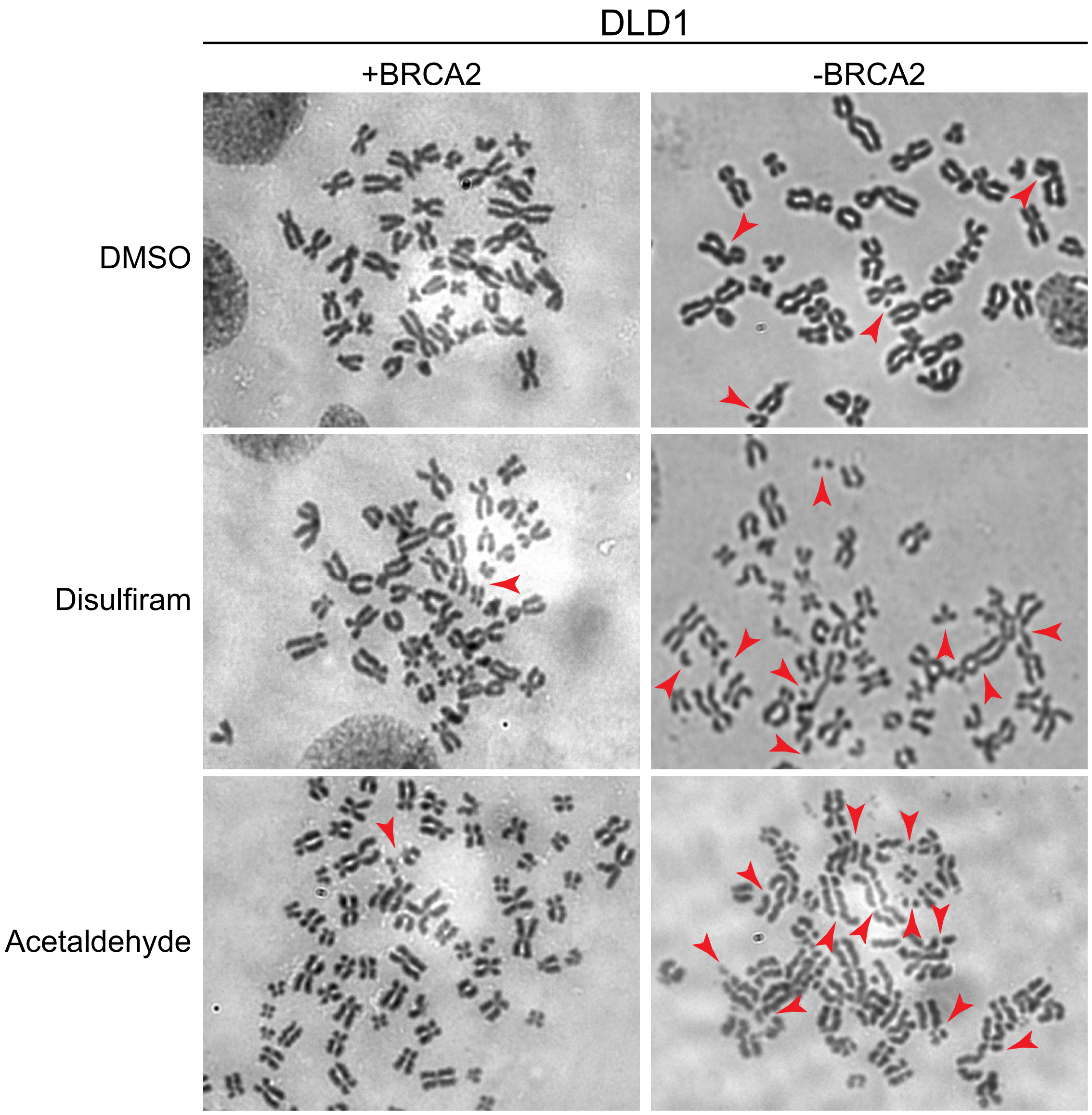

Figure EV4. Acetaldehyde and disulfiram treatments induce high levels of DNA breaks and chromosome rearrangements in BRCA2-deficient DLD1 cells.

Representative images of Giemsa-stained metaphase spreads prepared from BRCA2-proficient or BRCA2-deficient human DLD1 cells treated with 10 μM disulfiram or 4 mM acetaldehyde for 4 days, followed by overnight incubation with colcemid. Red arrowheads point to aberrations.

 © 2017 The Authors

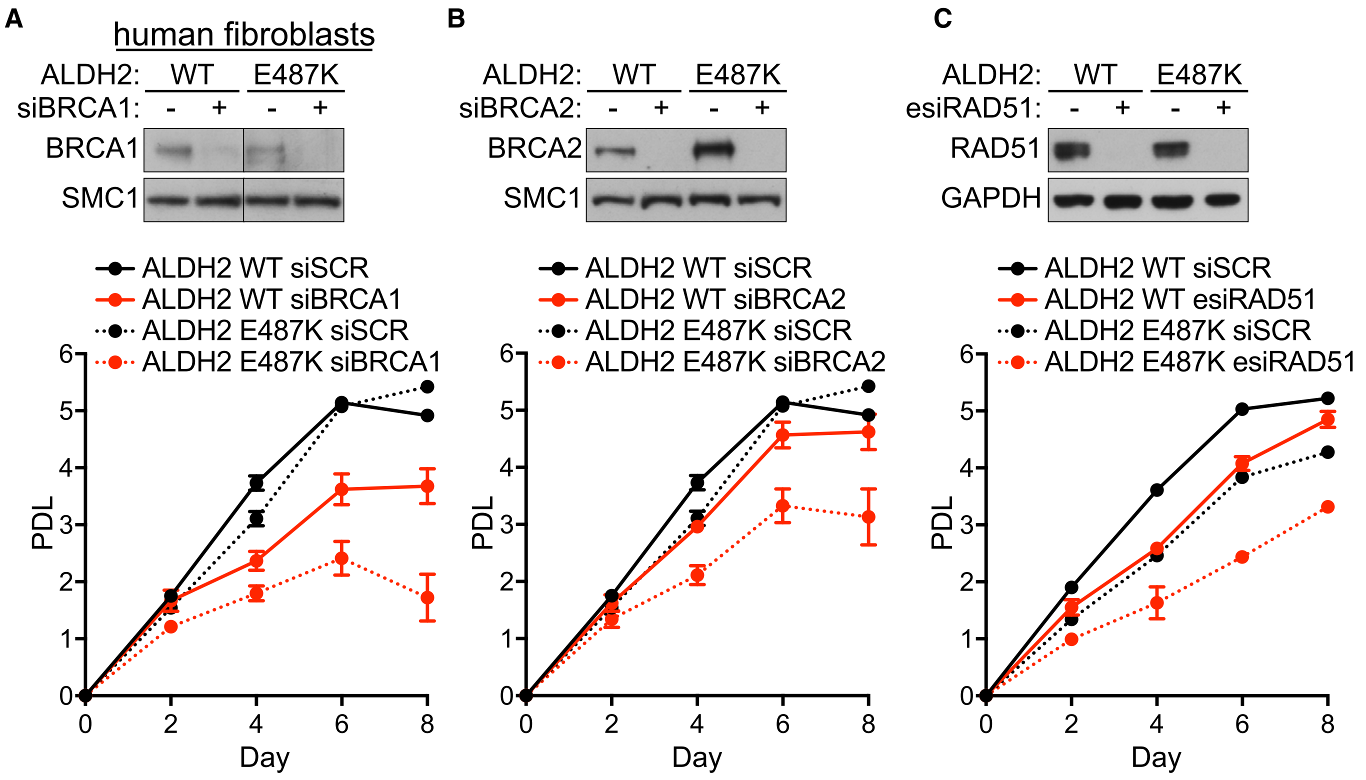

Figure EV5. Decreased proliferation rates in ALDH2-deficient human fibroblasts upon HR abrogation.

A–C Human fibroblasts carrying either WT ALDH2 or the E487K homozygous ALDH2 mutation were transfected with control (SCR) or siRNAs against BRCA1 (A), BRCA2 (B), or RAD51 (C). Cells were processed for proliferation assays 48 h after transfection. siRNAs were retransfected at an interval of 4 days. Graphs are representative of three independent experiments, each performed with three technical replicas. Error bars represent SD of three technical replica values obtained from a single experiment. Cell extracts were prepared 48 h after transfection and immunoblotted as shown. SMC1 or GAPDH were used as loading controls.
