# Supplementary figures and images for: BRCA1 and BRCA2 tumor suppressors protect against endogenous acetaldehyde toxicity
Source: EMBO Mol Med. 2017 Jul 20;9(10):1398–414. doi: 10.15252/emmm.201607446 (PMC5623864; doi:10.15252/emmm.201607446)

Figure 4D

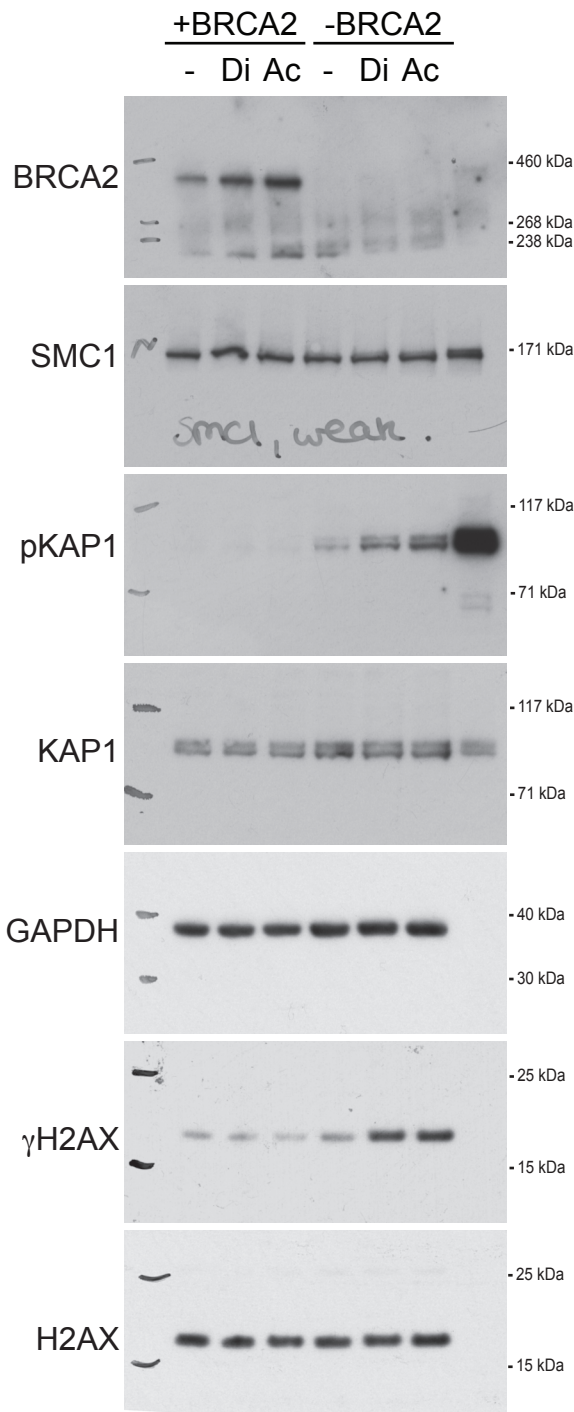

Figure 4E

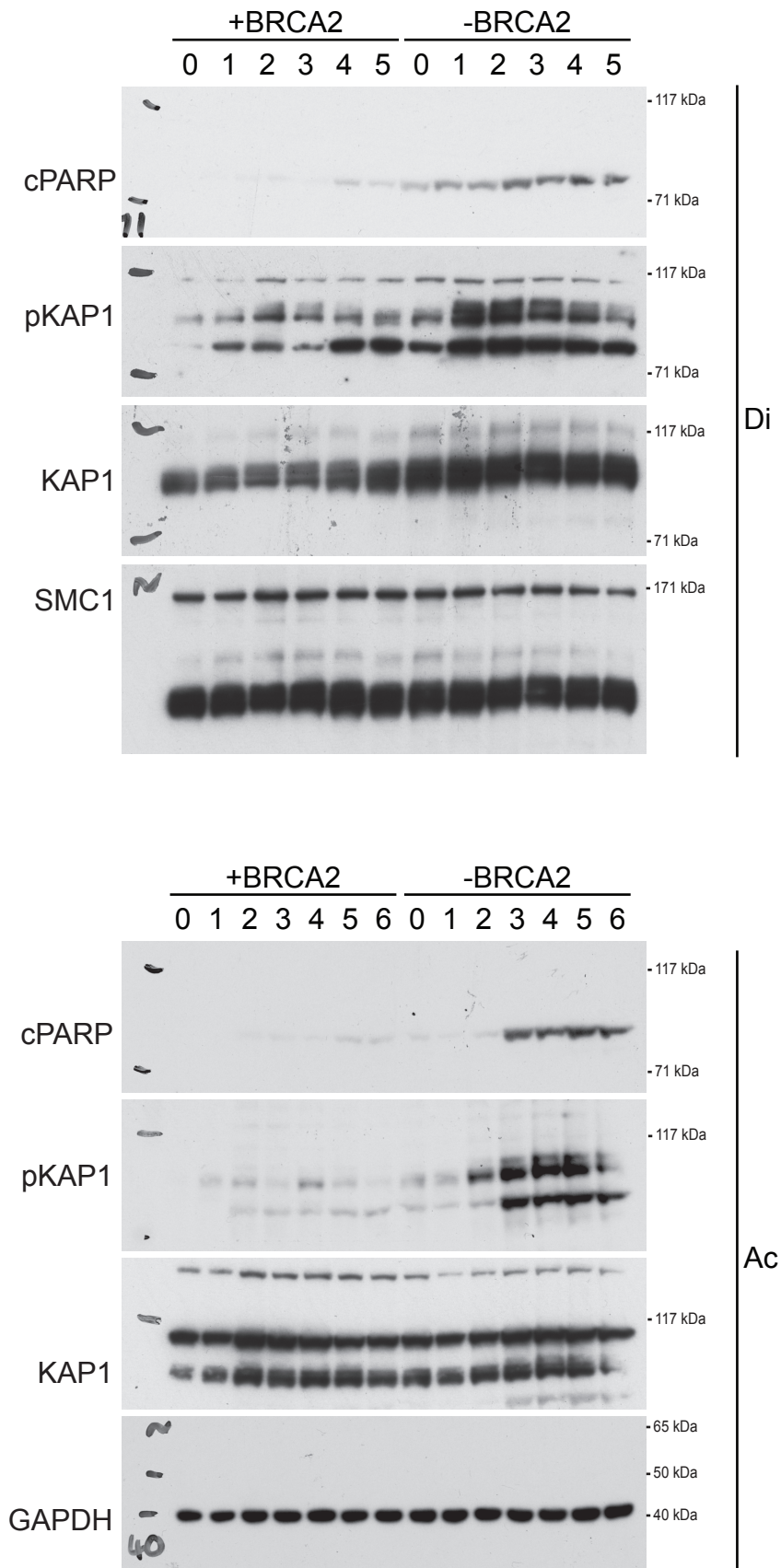

Supplement: Supplementary file 4 — Source Data for Figure 4 [file EMMM-9-1398-s003.pdf]

Figure 5C

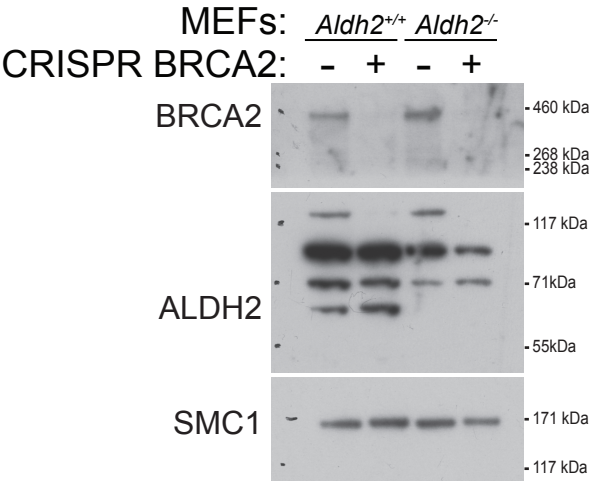

Figure 5E

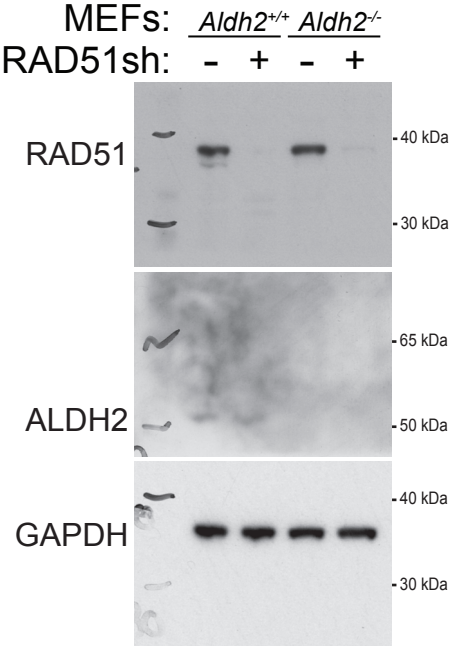

Supplement: Supplementary file 5 — Source Data for Figure 5 [file EMMM-9-1398-s004.pdf]
